# Exclusion of women from COVID-19 studies harms women's health and slows our response to pandemics

**DOI:** 10.1186/s13293-022-00435-1

**Published:** 2022-06-08

**Authors:** R. Craig Stillwell

**Affiliations:** Lexington, KY USA

**Keywords:** Sexual dimorphism, COVID-19, Gender differences, Public health

## Abstract

Sex and gender inclusion are crucial in bringing COVID-19 to an end and preventing the next pandemic. Despite this, almost all research studies on COVID-19 and clinical trials of vaccines do not include data on women. How can we combat the pandemic if half of the human population is left out of COVID-19 research? The life-long consequences of this neglect could be severe for women all over the world, particularly with the emergence of new variants that could exaggerate sex differences even further. Here I review recent studies and argue that taking a gender/sex approach to the study of this pandemic would expedite its end and improve the general health of women in substantial ways.

## Highlights

- It is now established that men and women respond differently to infection from COVID-19 and that clinical outcomes from this infection are more severe in men.
- Studies still neglect to include more women in clinical trials despite substantial differences between sexes in these outcomes.
- Neglecting to include women or enough women in research studies on COVID-19 can lead to recommendations that are not appropriate for women or even recommendations that harm their health.
- Pandemic research and medical research in general could be transformed by focusing more on sex-specific effects of disease and treatment.

## Background

We know that men and women respond differently to COVID-19 infection and that substantially more men die from infections of the virus. However, we ignore sex differences in most current studies on COVID-19, and neglect to account for gender/sex in vaccine trials. The life-long consequences of this neglect could be severe for women all over the world, especially with the appearance of new variants. I argue in my paper that taking a gender/sex approach to the study of this pandemic and medical studies, in general, could revolutionize the healthcare industry.

## Main text

Gender bias is embedded in medical research, from inception to discovery to understanding, to application, and every step in between. Studies of medicine in the US reveal disparities in funding, research publications, medical diagnoses and healthcare quality across genders. Recent investigations demonstrate how the exclusion of women from clinical trials, and sometimes the complete absence of women, contributes to the under-representation of women in medical and healthcare research [1]. Efforts have been made to improve the inclusion of women in medical studies. However, these improvements and changes to the status quo are not universal because persistent barriers and obstacles remain that hinder the advancement of women's health. One of these obstacles is the exclusion of women from COVID-19 clinical trials, a vital component of current medical research. This is especially critical with the emergence of new variants that may exaggerate sex differences in infections and disease outcomes even further.

COVID-19 causes men to have more severe symptoms and a higher death rate than women. Initial small studies in China indicated that men tend to have a higher severity of disease than women and that male individuals with comorbidities were more likely to suffer severe outcomes, but similar associations were not observed in women [2, 3]. In addition, using data from major healthcare providers, a study found that men were more likely than women to be susceptible to SARS-CoV-2 and more likely to be hospitalized than most COVID-19 patients [4].

A landmark study found that male bias in COVID-19 mortality in 37 out of 38 countries that provided sex-specific data [5]. There is also evidence for sex differences in COVID-19 severity, with morbidity and mortality higher in men than in women [1]. The higher mortality rate among men with COVID-19 raises the question of whether men are more susceptible than women [6].

Sex differences in the immune system in viral infections can lead to differential regulation of innate and adaptive immune responses, which in turn regulate gender-specific pathogenesis and mortality by different pathogens[1, 6]

Males and females show marked differences in the immune system response, with females triggering a stronger immune response to pathogens. These differences are a major factor in viral load, disease severity and mortality. Males and females show differences in the immune response to the challenge of viral infection [6].

Men and women differ in innate and adaptive immune responses, which may be partly related to sex-specific inflammatory reactions resulting from X-chromosomal inheritance. Both men and women are known to react differently to foreign self-genes, and sex-specific differences in the immune response have been documented [7]. Furthermore, differences in the sex hormone environment can be critical for a viral infection, as estrogen has immune-boosting and testosterone immunosuppressive effects [2, 6, 8].

These X chromosomes contain a higher density of X-related genes, and women have stronger innate and adaptive immune responses than men [6]. Sex-specific disease outcomes and viral infections can be attributed to the sex-dependent production of steroid hormones, the differences in the copy number of X-linked immune responses genes, or the presence of disease susceptibility genes. These differences could contribute to X chromosome genes for sex hormones such as estrogen, progesterone and androgens [1].

Males and females differ in their susceptibility to viral infections and their response to them, resulting in sex-specific differences in frequency and severity of the disease [2, 9]. The reduced susceptibility of women to viral infections is attributed to the protection of the X chromosome, and sex hormones play an important role in innate and adaptive immunity [1]. SARS-CoV-2 uses the ACE2 endothelial entry receptor gene (ACE2) on the X chromosome, which is the reason for the higher prevalence of COVID-19 in men than in women. [10]

Other immune cells such as natural killer cells and macrophages exhibit differentiated gene expression according to sex, which could help explain the stronger immune response of women. PDCs and other immune cells are regulated by sex hormones, and the more mature phenotype is the one that responds better to antiviral pathways in women than in men. It has been reported that sex differences in the innate and adaptive immune systems are responsible for the female advantage of COVID-19 [7, 8].

Although studies have shown that antibody production in men tends to be lower in many viral infections and vaccines than in women, data on sex differences in antibody responses to SARS-CoV-2 are inconsistent. Due to the pervasive nature of gender-specific data, some have argued that there are no real biological differences in responses to the virus between the sexes [8, 11].

Previous reports have described fundamental differences between sexes in the immune response to infections, including a robust innate antiviral interferon response and increased adaptive immunity to viral antigens in women. These differences in people with SARS-CoV-2 are likely to lead to more effective virus control in women, which can contribute to a lower risk of developing serious diseases. Extensive data show that there are sex differences in the proportion of people infected with the virus, with men at higher risk of serious illness and death than women [11].

Differences in susceptibility to respiratory infectious diseases between males and females have been shown in rodent models. In COVID-19 patients, it is expected that hitherto undetected differences in disease presentation and progression between men and women can influence the severity of the viral infection, the course of the disease, and the side effects of initiating therapy. The main findings of this study should be interpreted in the context of a large body of evidence showing sex differences in COVID-19 survival [5, 12].

This seems to explain the high ratio between men and women in this study, but does not justify including more than twice as many men as women in assessing the treatment of a novel disease with different risks. Less attention has been paid to biological sex differences and their impact on COVID-19 results [5, 10].

This article summarizes the available literature on proposed molecular and cellular markers of COVID-19 infection, their association with health outcomes and reported changes by gender. Except for a small observational study conducted in China, which examined potential sex differences in COVID-19 infections, research on this important topic has remained limited in the United States [2].

## Conclusions

Coronavirus disease (COVID-19) (SARS-CoV-2), is a global public health problem with profound effects on most aspects of social well-being, including physical and mental health. A wealth of studies suggest that there is a sex disparity in the severity and outcome of COVID-19 patients, the mechanism of viral infection, the immune response to the virus and the development of systemic inflammation and resulting systemic complications (e.g., thromboembolism). Epidemiological data show that there is no sex difference in severity of the disease, the favorable course of the virus in women compared to men and the age at which the rate of SARS-CoV-2 infection appears to be similar between the sexes [1]. Nevertheless, including women in COVID-19 studies is essential to understanding whether the virus has both short-term and long-term consequences for women's health, particularly with the appearance of new variants that have unforeseen consequences on sex disparities in COVID-19 outcomes.

## Data Availability

Everything is contained in this manuscript.
